# Mosquito Attraction: Crucial Role of Carbon Dioxide in Formulation of a Five-Component Blend of Human-Derived Volatiles

**DOI:** 10.1007/s10886-015-0587-5

**Published:** 2015-05-31

**Authors:** Joop J. A. van Loon, Renate C. Smallegange, Gabriella Bukovinszkiné-Kiss, Frans Jacobs, Marjolein De Rijk, Wolfgang R. Mukabana, Niels O. Verhulst, David J. Menger, Willem Takken

**Affiliations:** Laboratory of Entomology, Wageningen University, P.O. Box 16, 6700 AA Wageningen, The Netherlands; Laboratory of Genetics, Wageningen University, P.O. Box 309, 6700 AH Wageningen, The Netherlands; International Centre of Insect Physiology and Ecology, P.O. Box 30772–00100 GPO, Nairobi, Kenya; School of Biological Sciences, University of Nairobi, P.O. Box 30197–00100 GPO, Nairobi, Kenya

**Keywords:** Olfaction, Behavioral disruption, Kairomone, Trapping, Carbon dioxide, *Anopheles gambiae*, Butan-1-amine, 3-methyl-1-butanol

## Abstract

Behavioral responses of the malaria mosquito *Anopheles coluzzii* (*An. gambiae sensu stricto* molecular ‘M form’) to an expanded blend of human-derived volatiles were assessed in a dual-port olfactometer. A previously documented attractive three-component blend consisting of NH_3_, (*S*)-lactic acid, and tetradecanoic acid served as the basis for expansion. Adding 4.5 % CO_2_ to the basic blend significantly enhanced its attractiveness. Expansion of the blend with four human-derived C4-volatiles was then assessed, both with and without CO_2_. Only when CO_2_ was offered simultaneously, did addition of a specific concentration of 3-methyl-1-butanol or 3-methyl-butanoic acid significantly enhance attraction. The functional group at the terminal C of the 3-methyl-substituted C4 compounds influenced behavioral effectiveness. In the absence of CO_2_, addition of three concentrations of butan-1-amine caused inhibition when added to the basic blend. In contrast, when CO_2_ was added, butan-1-amine added to the basic blend strongly enhanced attraction at all five concentrations tested, the lowest being 100,000 times diluted. The reversal of inhibition to attraction by adding CO_2_ is unique in the class Insecta. We subsequently augmented the three-component basic blend by adding both butan-1-amine and 3-methyl-1-butanol and optimizing their concentrations in the presence of CO_2_ in order to significantly enhance the attractiveness to *An. coluzzii* compared to the three- and four-component blends. This novel blend holds potential to enhance malaria vector control based on behavioral disruption.

## Introduction

Mosquitoes belong to a group of blood-feeding insects that contribute to transmission of serious infectious diseases like dengue, malaria, chikungunya, and Rift Valley fever. Among these, malaria mosquitoes in the genus *Anopheles* are the most important species because of their high prevalence and incidence of infection with malaria parasites, *Plasmodium* spp. (WHO 2013). Although significant reductions in malaria have been achieved in the last decade, mainly through the Roll Back Malaria program of the World Health Organization, further progress in malaria control can be achieved by rational and novel strategies to add to the armament of existing tools (Alonso et al. 2011).

Anopheline mosquitoes feed on humans as blood hosts, enabling the transmission of *Plasmodium* parasites from infected to uninfected hosts. Vector-host contact is achieved through chemoreception of volatile cues emitted by the blood host (Zwiebel and Takken 2004). Host volatiles are perceived by olfactory organs located on the head of the mosquito, in particular the antennae and maxillary palps (Qiu and Van Loon 2010). In recent years, the molecular basis of olfactory perception of mosquitoes has been elucidated by the discovery of a suite of olfactory receptor (OR) genes that recognize volatile host cues (Carey et al. 2010; Liu et al. 2010). Binding of the host-derived volatile organic molecules to ORs trigger signal transduction in olfactory receptor neurons, which transmit electrophysiological activity to the olfactory lobe in the brain ultimately leading to a behavioral response (Qiu and Van Loon 2010). Recently, significant progress has been made in the identification of a number of these odorants, resulting in the creation of odor blends that are as attractive as a human host (Menger et al. 2014; Mukabana et al. 2012; Okumu et al. 2010). These blends have been formulated during an iterative process involving molecular, physiological, and behavioral assays on anopheline mosquitoes *in vitro* and *in vivo* (Carey et al. 2010; Carlson and Carey 2011; Qiu et al. 2011; Rinker et al. 2012; Smallegange et al. 2010, 2012).

In the present study, we re-assessed the role of carbon dioxide in attraction of the African malaria mosquito *An. coluzzii* Coetzee & Wilkerson sp. n. (renamed from *An. gambiae sensu stricto* molecular ‘M-form’; Coetzee et al. 2013) to odor blends composed of C4-compounds, which have previously been reported to be attractive or inhibitory in the absence of carbon dioxide (Smallegange et al. 2012; Verhulst et al. 2011a). This finding led us to augment the three-component blend of ammonia, lactic acid, and tetradecanoic acid that we reported before as an effective kairomone blend mimicking the attraction of human subjects (Smallegange et al. 2009, 2012) with butan-1-amine and 3-methyl-1-butanol, a volatile produced by microbiota on the human skin (Verhulst et al. 2009, 2011a).

## Methods and Materials

### Mosquitoes

The female mosquitoes used for the laboratory experiments were collected from the colony of the Suakoko strain of *An. coluzzii* reared at Wageningen University, The Netherlands, where it has been reared on human blood from 1988 onward. Larvae were kept in tap water and fed on Tetramin® fish food (Tetrawerke, Melle, Germany). Pupae were collected daily and transferred to cubic gauze cages (30 × 30 × 30 cm) for emergence. Adult mosquitoes were kept under an 12 / 12 h L/D photo:scotophase at 27 ± 1 °C and 80 ± 5 % relative humidity (RH), and provided with a 6 % glucose solution on filter paper *ad libitum*.

### Tested Compounds

We tested four C4-compounds functionalized at the 1- and/or 3-positions: 3-methylbutanoic acid (*syn*. isovaleric acid; Sigma-Aldrich; 99 % pure), 3-methyl-1-butanol (Fluka, 99.8 %), 3-methylbutanal (Fluka, 98 %), and butan-1-amine (Sigma-Aldrich, 99 %). The first three are produced by human skin-associated bacteria that use fatty acids produced by eccrine sweat glands as substrates (James et al. 2004; Verhulst et al. 2011a,b). The amine occurs in human effluvia and feces (Ellin et al. 1974; Smith and Macfarlane 1996). Each compound was added individually to a three-component basic blend, consisting of ammonia, (*S*)-lactic acid, and tetradecanoic acid (Table 1; Smallegange et al. 2005), to test their effect on the degree of attractiveness of the reference blend (Smallegange et al. 2009; Verhulst et al. 2010, 2011a). The resulting blends were tested either as such or augmented with CO_2_ since this compound has been found essential to evoke a behavioral response in (semi-)field settings (Njiru et al. 2006; Schmied et al. 2008). To guarantee a constant emission rate of the individual compounds within and between experiments, all compounds (except CO_2_) were dispensed individually from low density polyethylene (LDPE) sachets (Audion Elektro, The Netherlands; Smallegange et al. 2012; Torr et al. 1997) with a sealed area of 25 × 25 mm, and variable sheet thickness (thickness stated in Table 1 for the three components of the basic blend; 0.20 mm was used for the C4-compounds). The sachets were suspended from metal hooks that were placed inside the trapping devices in the olfactometer (Smallegange et al. 2012; Verhulst et al. 2010)

**Table 1:** Composition of the attractive basic blend used in the olfactometer experiments

Compound	Concentration (% in w/w)	LDPE sheet thickness (mm)	Solvent
Ammonia	25	0.03	Distilled water
(*S*)-Lactic acid	88–92	0.05	Distilled water
Tetradecanoic acid	>99	0.03	(Solid)

### Olfactometer Experiments

In the laboratory, a three-layer olfactometer was used, consisting of three polycarbonate flight chambers (each 1.50 × 0.50 × 0.50 m) on top of each other, allowing us to run three experiments simultaneously (Smallegange et al. 2012; Verhulst et al. 2010). Mosquito rearing, preparation and the performance of the olfactometer experiments, as well as cleaning of the olfactometer parts, were done as described in Smallegange et al. (2012). The air flowing into the flight chambers was maintained at a relative humidity above 70 % and had an average temperature of 26.6 ± 1.1 °C. Data loggers (type MSR1455, MSR Electronics GmbH, Switzerland) mounted inside the flight chambers measured an average temperature and relative humidity of 26.7 ± 0.8 °C and 62.7 ± 5.9 %, respectively. Each blend was tested six to eight times, on different days. The sequence in which different concentrations of a compound were tested was randomized on the same day and between days, and between the three flight chambers. Controls and treatments were alternated between right and left ports in different replicates to rule out any positional effects. Experiments with clean air only in either port were done to confirm the symmetry of the trapping systems (data not shown). Air containing 4.5 % CO_2_ was released at 250 ml/s from glass pipette tips, placed inside the flight chamber at 5 cm in front of each olfactometer port (Spitzen et al. 2008).

### Optimization of an Attractive Blend Containing Butan-1-amine and 3-Methyl-1-butanol

Additional tests were designed to determine the effect of adding butan-1-amine and 3-methyl-1-butanol, at a range of concentrations in paraffin oil, to the basic blend in the presence of CO_2_. Butan-1-amine was added to blends containing various dilutions of 3-methyl-1-butanol to assess whether certain combinations would be more attractive than blends without butan-1-amine.

### Statistical Analysis

For each two-choice olfactometer experiment a *χ*^*2*^-test was used to test whether the distribution of the total number of mosquitoes caught in the treatment or control trapping device over all replicates differed from a 50:50 distribution at *α* = 0.05.

## Results

### Effect of C4-Compounds on Attractiveness of the Three-Compound Basic Blend (NH_3_ + LA + C14) with or without CO_2_

**Fig. 1:**
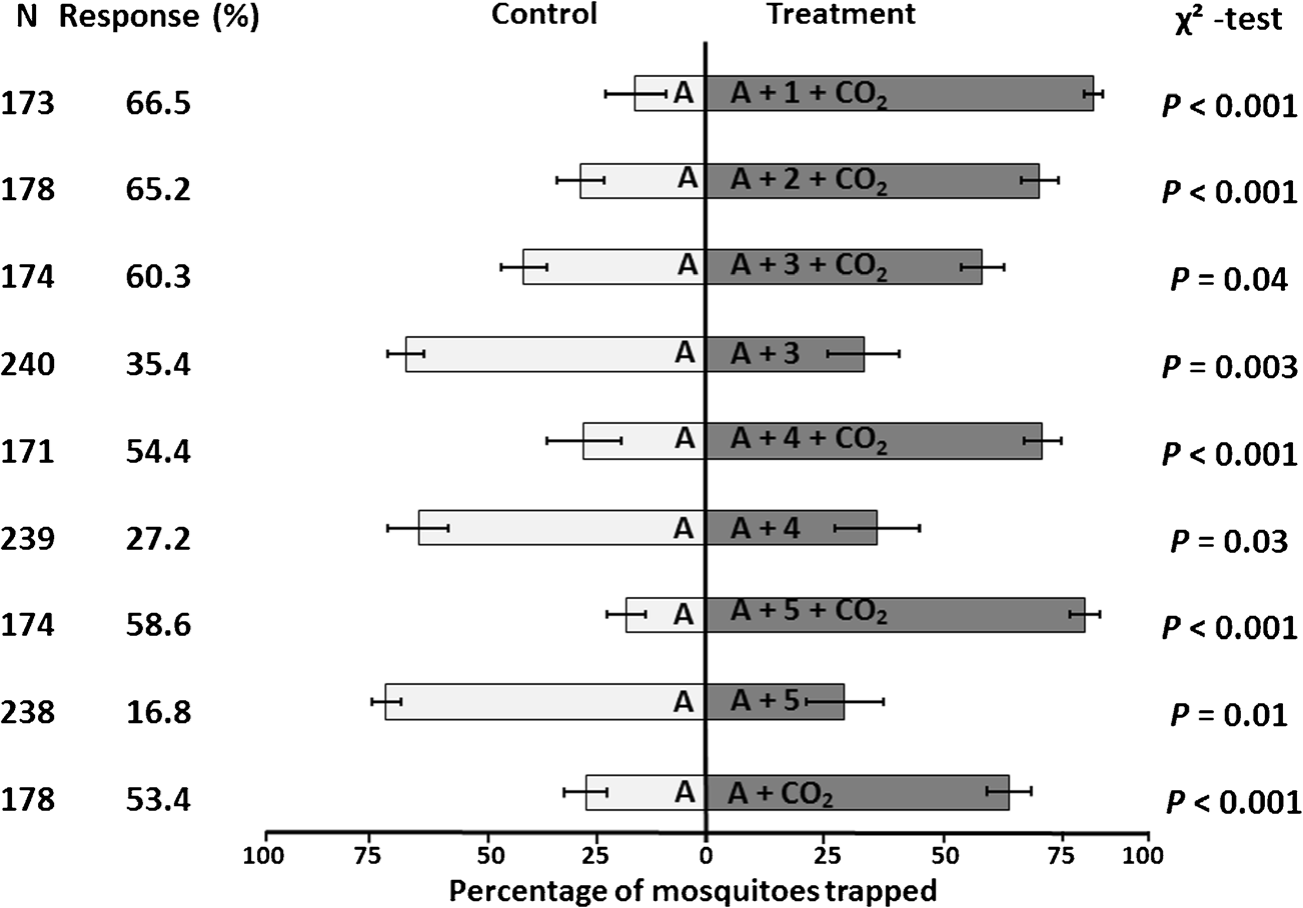
Reversal of repellence to attraction of *Anopheles coluzzii* by butan-1-amine depending on addition of carbon dioxide. Mean percentages of released mosquitoes that were trapped with the respective odor blends. Error bars represent SEM. *N* is the total number of mosquitoes that were introduced in the olfactometer, six to eight groups of 30 females minus the ones that did not leave the release cage. The six to eight bioassays were performed on at least three test days. The response is the percentage that was trapped by the end of the 15-min experiment. The numbers placed inside the bars indicate the compound(s) and concentrations that were added to the basic blend consisting of NH_3_, (*S*)-lactic acid, and tetradecanoic acid. Mean percentages of mosquitoes that were trapped when exposed to the basic blend (A) augmented with butan-1-amine in five concentrations, with or without carbon dioxide. *P*-values are based on *χ* ^*2*^-tests. Data for four-component blend without carbon dioxide were taken from Smallegange et al. (2012) and reproduced here for direct comparison. A = basic blend; 1: 0.001 % butan-1-amine; 2: 0.004 %; 3: 0.01 % butan-1-amine; 4: 0.1 % butan-1-amine; 5: 1.0 % butan-1-amine

Adding 3-methyl-1-butanol (at 0.01 %) or 3-methylbutanal (at 1 %) resulted in significantly enhanced attraction in the absence of CO_2_, whereas 3-methylbutanoic acid was ineffective at the three concentrations tested (Table 2; data on four-component blends without CO_2_ taken from Verhulst et al. (2011a)). In contrast, adding butan-1-amine to the basic blend in the absence of CO_2_ inhibited the trap entry response at all three concentrations tested (Fig. 1; data on four-component blends without CO_2_ taken from Smallegange et al. (2012)).

**Table 2:** Response of *Anopheles coluzzii* to three bacterial C4-compounds tested in a dual-choice olfactometer in three concentrations in low density polyethylene (LDPE) sachets in the presence of the three-component basic blend consisting of NH_3_, (*S*)-lactic acid, and tetradecanoic acid, with (+) or without (−) carbon dioxide. Control = basic blend (−) or basic blend + carbon dioxide (+). Data for four-component blends without carbon dioxide were taken from Verhulst et al. (2011a) and reproduced here for direct comparison. Values for control and treatment refer to the numbers of mosquitoes caught out of the total number that left the release cage (*N*) over six replicated tests (*N*_*max*_ = 180). Percentages refer to overall response. *P*-values are based on *χ* ^*2*^-tests

Compound added to basic blend	4.5 % CO_2_	0.01 %		0.1 %		1 %	
		Control	Treatment	Control	Treatment	Control	Treatment
3-methyl-1-butanol	−	25	41	28	20	31	29
		*P* = 0.048 (*N* = 157, 42 %)		*P* = 0.25 (*N* = 158, 30 %)		*P* = 0.80 (*N* = 163, 37 %)	
	+	37	43	25	48	26	27
		*P* = 0.50 (*N* = 174, 46 %)		*P* = 0.007 (*N* = 175, 42 %)		*P* = 0.89 (*N* = 171, 31 %)	
3-methylbutanal	−	16	16	21	24	18	34
		*P* = 1.0 (*N* = 172, 19 %)		*P* = 0.65 (*N* = 158, 28 %)		*P* = 0.03 (*N* = 170, 31 %)	
	+	49	46	63	34	42	56
		*P* = 0.76 (*N* = 169, 56 %)		*P* = 0.003 (*N* = 176, 55 %)		*P* = 0.16 (*N* = 178, 55 %)	
3-methylbutanoic acid	−	18	30	24	33	25	22
		*P* = 0.08 (*N* = 161, 30 %)		*P* = 0.23 (*N* = 161, 35 %)		*P* = 0.66 (*N* = 163, 29 %)	
	+	56	52	37	61	47	34
		*P* = 0.23 (*N* = 171, 68 %)		*P* = 0.02 (*N* = 175, 56 %)		*P* = 0.15 (*N* = 174, 47 %)

A significant increase in attractiveness compared to that exerted by the basic blend alone was observed when CO_2_ was added (Fig. 1). In the presence of CO_2_, adding 3-methyl-1-butanol or 3-methylbutanoic acid at 0.1 % significantly increased the attractiveness of the basic blend (*P* = 0.007 and *P* = 0.02, respectively). A reverse effect was found for 3-methylbutanal, the addition of which significantly inhibited the response to the basic blend in the presence of CO_2_ at 0.1 % (Table 2). Remarkably, adding butan-1-amine in the presence of CO_2_ completely reversed the inhibitory effect of this compound to strong attraction at the three concentrations tested. Significantly higher attractiveness was preserved after diluting the lowest of the three concentrations of butan-1-amine by a factor of 10 and 100 (Fig. 1).

The overall response rate (expressed as the percentage of all released mosquitoes entering either trap) ranged from 17 to 42 % in the absence of CO_2,_ and from 31 to 68 % in the presence of CO_2_. The highest values were found for butan-1-amine (59–67 %) and 3-methylbutanoic acid (47–68 %).

### Formulation of a Five-Component Attractive Blend Containing Butan-1-amine and 3-Methyl-1-butanol

**Fig. 2:**
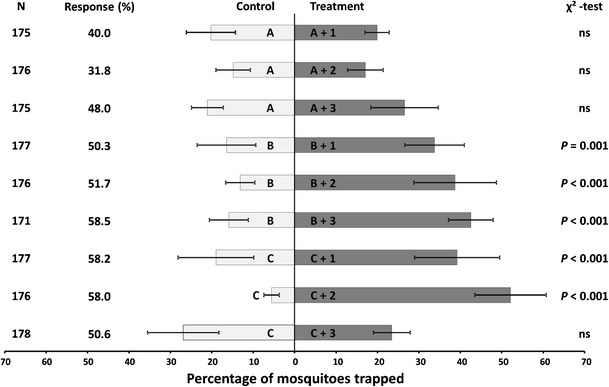
Olfactory bioassays testing different blend ratios of 3-methyl-1-butanol and butan-1-amine. Mean percentages of released mosquitoes that were trapped with the respective odor blends. Error bars represent SEM. *N* is the total number of mosquitoes that were introduced in the olfactometer, six groups of 30 females minus the ones that did not leave the release cage. The six bioassays were performed on at least three test days. The response is the percentage that was trapped by the end of the 15-min experiment. *P*-values are based on *χ* ^*2*^-tests. The letters and numbers placed inside the bars indicate the compound(s) and concentrations that were added to the basic blend consisting of NH_3_, (*S*)-lactic acid, and tetradecanoic acid and 4.5 % CO_2_: A = 0.1 % 3-methyl-1-butanol; B = 0.01 % 3-methyl-1-butanol; C = 0.001 % 3-methyl-1-butanol; 1 = 0.01 % butan-1-amine; 2 = 0.004 % butan-1-amine; 3 = 0.001 % butan-1-amine

In the presence of carbon dioxide, particular concentrations of 3-methyl-1-butanol, 3-methylbutanoic acid, and all five concentrations of butan-1-amine increased the attractiveness of the basic blend. Due to the malodor associated with 3-methylbutanoic acid, we discarded it for further tests (Okumu et al. 2010). We subsequently tested which concentrations of 3-methyl-1-butanol and butan-1-amine and their combinations significantly augmented the attractiveness of the basic blend in the presence of CO_2,_ with the aim to find the lowest active concentrations (Fig. 2). When the basic blend was prepared with 0.01 % 3-methyl-1-butanol, the addition of 0.01, 0.004, or 0.001 % butan-1-amine resulted in higher trap catches (*χ*^*2*^-test, *P* ≤ 0.001). When 0.001 % 3-methyl-1-butanol was added to the basic blend, the addition of 0.01 or 0.004 % butan-1-amine resulted in a higher number of trapped mosquitoes (*χ*^*2*^-test, *P* < 0.001). Other combinations did not lead to significant preferences for either one of the blends.

## Discussion

### Carbon Dioxide: An Essential Component of Host-derived Kairomone Blends

In a previous study we tested the effect of butan-1-amine on the behavioral response of *An. coluzzii*. We found that in the absence of CO_2_ significantly more mosquitoes were caught in the port with the three-component basic blend than with this blend to which butan-1-amine at concentrations of 0.01, 0.1, and 1.0 % had been added (Smallegange et al. 2012), suggesting an inhibitory effect caused by butan-1-amine. In the laboratory assays that we have employed thus far, CO_2_ was not needed to demonstrate kairomonal effects of volatile compounds with this anopheline species (Smallegange et al. 2005, 2009, 2012), and data presented here corroborate this. However, in order to mimic the standard trapping method in (semi-)field test conditions more closely (Mukabana et al. 2012), in the present study, CO_2_ was added to the basic blend in the laboratory olfactometer bioassays. This resulted in a significantly enhanced attractiveness and overall increase of the response rate. Unexpectedly, butan-1-amine, added to the basic blend + CO_2_, evoked strong attraction of *An. coluzzii.* Indeed, progressive dilutions of butan-1-amine to 1:100,000 elicited a highly significant preference, with trap entry responses ranging between 65.2 and 66.5 % (Fig. 1). To the best of our knowledge, the remarkable reversal of the behavioral effect of butan-1-amine that depends on the presence of CO_2_ is unique in insect chemical ecology and highlights a crucial role in the kairomone blend. Geier and Boeckh (1999) reported that both CO_2_ and (*S*)-lactic acid were attractive on their own to *Ae. aegypti* (L.), and that their combination exerted a more than additive attraction. More generally, both activating and attractant roles have been ascribed to CO_2_ in the host-seeking behavior of mosquitoes and other hematophagous insects (Gillies 1980; McMeniman et al. 2014; Mboera and Takken 1997). Small fluctuations in carbon dioxide concentrations recently have been demonstrated to increase the frequency of landing behavior on human-odor treated gauze (Webster et al. 2015).

The ionotropic (IR) family of olfactory receptors now has been identified (Benton et al. 2009; Guo et al. 2014), expanding the range of olfactory receptors to include those binding more polar molecules, such as butan-1-amine and 3-methylbutanoic acid. Aliphatic carboxylic acids, many of which are not binding to ORs, appear to be agonists of IRs in *Drosophila melanogaster* (Abuin et al. 2011). We previously have shown the importance of carboxylic acids in the behavior of anopheline mosquitoes (Smallegange et al. 2005, 2009; Okumu et al. 2010). These compounds are not among the agonists of the AgOR family of olfactory receptors (Carey et al. 2010), but are ligands of the AgIR family (Liu et al. 2010; Rinker et al. 2012) as well as to gustatory receptors (Kwon et al. 2006). Several carboxylic acids, amines and ammonia evoke a response in IRs of *D. melanogaster* (Abuin et al. 2011; Min et al. 2013). By contrast, 3-methyl-1-butanol, a compound that activates eight AgOR receptors (Carey et al. 2010), evokes a strong behavioral response in *An. gambiae s.s*. ‘S form’ (Carey et al. 2010; Mukabana et al. 2012; Verhulst et al. 2011a). The effect of the three 3-methyl-substituted C4 compounds with either an aldehyde-, alcohol-, or carboxylic acid-group at the terminal C differs qualitatively, pointing to strict structural requirements for activity.

Electrophysiological studies on *An. coluzzii* have reported responses to ammonia, butan-1-amine, lactic acid, and 3-methylbutanoic acid in olfactory receptor neurons in antennal grooved peg sensilla (Qiu et al. 2006). However, responses to 3-methyl-1-butanol and 3-methylbutanal have been detected in sensilla trichodea of response type TE1 and in capitate peg neurons (Qiu et al. 2006; Suer 2011). Neurons responding to CO_2_ reside in capitate peg sensilla on the maxillary palp in *An. gambiae sensu stricto* ‘S form’ (Lu et al. 2007). Recently, McMeniman et al. (2014) demonstrated the significance of the CO_2_ receptor on the maxillary palp of the mosquito *Aedes aegypti*. RNAi-mediated silencing of this receptor showed that mosquitoes could no longer recognize their human host from a distance. ORNs in sensilla trichodea and grooved pegs project to different glomerular areas of the antennal lobe, the first integration center in the deutocerebrum of the brain (Anton et al. 2003; Anton and Rospars 2004). Carbon dioxide sensitive neurons contained in sensilla on the maxillary palp also project to the antennal lobe, but to a distinctly different area (Anton et al. 2003). We note that olfactory receptors of all three known classes, i.e., ORs, IRs, and GRs, are involved in the olfactory perception of CO_2_ and the five-component blend presented here.

### Formulation of a Five-Component Odor Blend That Exhibits Increased Attractiveness

Our results demonstrate that the synergistic three-component blend of ammonia, (*S*)-lactic acid, and tetradecanoic acid that we developed previously (Smallegange et al. 2005, 2009, 2012) can be made significantly more attractive by addition of two other compounds, butan-1-amine, increasing attractiveness synergistically when CO_2_ is added, and 3-methyl-1-butanol. Mweresa (2014) validated the results on the five-component blend we report here based on olfactometer assays augmented with CO_2_, under semi-field and field conditions in Kenya. Although Mweresa (2014) used nylon strips instead of LDPE material as odor-dispensing material, the data suggest that in both cases it was the combined effect of the five-component blend + CO_2_ that induced the observed attraction response in *An. gambiae sensu lato*.

The strong and consistent responses of *An. gambiae sensu lato* to the five-component blend augmented with CO_2_ under field conditions suggests that the blend contains essential compounds in specific ratios, which these mosquitoes use for recognition of human hosts. Of these five compounds, (*S*)-lactic acid is the only compound that is unique to humans (Dekker et al. 2002), although we showed previously that (*S*)-lactic acid on its own is a poor attractant (Smallegange et al. 2005). Williams et al. (2006) reported similar findings for *Ae. aegypti*, which was attracted only to a blend of odorants in specific ratios. For the latter species, hexanoic acid appears a crucial compound (Bosch et al. 2000), whereas in *An. coluzzii*, it is tetradecanoic acid (Smallegange et al. 2005, 2009).

In Summary, we revealed a crucial role of CO_2_ in formulating a five-component blend that is significantly more attractive than a three-component blend. The five-component blend is characterized by particular ratios of the components. This blend holds promise for behavioral disruption of host-seeking mosquitoes (Menger et al. 2014; Mukabana et al. 2012; Okumu et al. 2010). It can be utilized to overcome the need to use human volunteers for the surveillance of malaria mosquitoes to monitor disease epidemiology, for mass trapping of malaria vectors, and contribute to reduced likelihood of mosquito biting.
